# Preventive Immunology for Livestock and Zoonotic Infectious Diseases in the One Health Era: From Mechanistic Insights to Innovative Interventions

**DOI:** 10.3390/vetsci12101014

**Published:** 2025-10-20

**Authors:** Eman Marzouk, Ahmed I. Alajaji

**Affiliations:** 1 Department of Public Health, College of Applied Medical Sciences, Qassim University, Buraydah 51452, Saudi Arabia; e.marzouk@qu.edu.sa; 2 Department of Veterinary Preventive Medicine, College of Veterinary Medicine, Qassim University, Buraydah 51452, Saudi Arabia

**Keywords:** preventive immunology, animal infectious diseases, One Health, vaccines, gene editing, antimicrobial resistance, diagnostics, public health

## Abstract

**Simple Summary:**

Infectious diseases in animals threaten livestock production, food safety, and human health. This review explains how preventive immunology is reshaping control strategies beyond traditional vaccines. We outline newer vaccine platforms such as mRNA and nanovaccines, along with complementary approaches including monoclonal antibodies, probiotics, bacteriophages, and CRISPR-based tools. We also highlight improved diagnostics and One Health surveillance as key for early warning and for managing antimicrobial resistance. Although regulatory, economic, and logistical hurdles remain, these advances have strong potential to reduce disease burden, limit antimicrobial use, and protect the health of animals, people, and ecosystems within sustainable food systems. We also discuss where and why these tools can fail (for example, gene-editing escape at high viral doses, phage host range and stability constraints, and strain-specific probiotic effects) and offer practical mitigations.

**Abstract:**

Preventive immunology is emerging as a cornerstone of animal infectious disease control within One Health, shifting emphasis from treatment to prevention. This review integrates mechanistic insights in host immunity with a comparative evaluation of next-generation interventions—mRNA/DNA and viral-vector vaccines, nanovaccines, monoclonal antibodies, cytokine modulators, probiotics/postbiotics, bacteriophages, and CRISPR-based approaches—highlighting their immunogenicity, thermostability, delivery, and field readiness. Distinct from prior reviews, we appraise diagnostics as preventive tools (point-of-care assays, biosensors, MALDI-TOF MS, AI-enabled analytics) that enable early detection, risk prediction, and targeted interventions, and we map quantifiable links between successful prevention and reduced antimicrobial use. We embed translation factors—regulatory alignment, scalable manufacturing, workforce capacity, equitable access in LMICs, and public trust—alongside environmental and zoonotic interfaces that shape antimicrobial resistance dynamics. We also provide a critical analysis of limitations and failure cases: gene editing may require stacked edits and concurrent vaccination; phage programs must manage host range, resistance, stability, and regulation; and probiotic benefits remain context-specific. Finally, we present a risk–benefit–readiness framework and a time-bound research agenda to guide deployment and evaluation across animal–human–environmental systems. Coordinating scientific innovation with governance and ethics can measurably reduce disease burden, curb antimicrobial consumption, and improve health outcomes across species.

## 1. Introduction

Infectious diseases in livestock and companion species remain a persistent threat to food security, international trade, and rural livelihoods, while also posing serious zoonotic risks to human populations [1]. The intensification of animal production, combined with global trade, urbanization, and climate variability, has created conditions that favor the emergence and re-emergence of infectious agents at the human–animal–ecosystem interface [2,3]. Within this context, preventive immunology is increasingly recognized as a cornerstone of sustainable health management in the One Health era [4].

The One Health Joint Plan of Action (2022–2026), launched by FAO, UNEP, WHO, and WOAH, emphasizes the urgency of strengthening prevention strategies through cross-sectoral surveillance, vaccination, and risk reduction at local, regional, and global levels [1]. Historical examples underscore the transformative impact of immunization, from the global eradication of rinderpest in 2011 to large-scale canine vaccination campaigns driving progress toward the “Zero by 30” rabies elimination target [5,6,7]. These successes demonstrate that coordinated prevention, when paired with surveillance and clear governance, can change the course of animal and human health.

Despite these achievements, growing pathogen diversity, ecological change, and the accelerating antimicrobial resistance (AMR) crisis underscore the limits of purely reactive approaches [8,9]. Recent surveillance and burden estimates continue to show substantial—and uneven—AMR pressures worldwide [10,11,12], underscoring the need to curb antimicrobial reliance. In this context, vaccination serves a dual purpose: it prevents infection and, as part of stewardship, helps reduce antimicrobial use. At the same time, emerging platforms such as mRNA and nanoparticle technologies are expanding opportunities for proactive control in veterinary species [13,14].

Next-generation vaccines—including recombinant protein, viral-vector, and messenger RNA (mRNA) platforms—paired with advanced nanoparticle adjuvants and delivery systems, enable faster development timelines, greater antigen specificity, and improved mucosal targeting. Together, these features can strengthen population-level (herd) protection [14,15,16,17,18]. Complementary preventive strategies—including monoclonal antibodies, bacteriophages, microbiome modulation, and modern diagnostics (e.g., POCT, biosensors, CRISPR assays, MALDI-TOF MS)—are increasingly integrated with data-driven analytics to enable early detection, strengthen surveillance, and support proactive, targeted interventions [19,20,21,22,23,24,25]. Together, they are reshaping surveillance from a reactive system into a proactive framework.

Gene-editing technologies such as CRISPR/Cas9 are being applied to generate disease-resistant livestock—illustrated by a calf engineered with reduced susceptibility to bovine viral diarrhea virus (BVDV)—and reviews confirm their expanding use across multiple species [26,27]. Synthetic biology approaches, including CRISPR-enabled modulation of rumen microbiota, are also emerging to simultaneously reduce methane emissions and enhance disease prevention [28].

However, these innovations raise ethical, regulatory, and societal challenges, particularly concerning oversight and public acceptance. For example, recent UK legislation restricts gene editing to crops, excluding farm animals, even though researchers advocate for its potential to improve animal health in the face of infectious and climate-driven threats [29]. International harmonization of regulatory frameworks will be essential to balance innovation with public trust. These developments reflect a paradigm shift from treatment-centered models toward prevention-driven, immunology-informed strategies that align with One Health priorities. This transition emphasizes prevention as the foundation of future resilience in both animal and human health [30].

From a One Health standpoint, preventing infection in livestock protects far more than animal productivity—it reduces zoonotic spillover and downstream human disease. The updated definition by the One Health High-Level Expert Panel explicitly links people, animals, and ecosystems within one prevention agenda [31]. History shows the public-health yield of animal-side action: the global eradication of rinderpest through coordinated veterinary vaccination delivered lasting gains for food security and community resilience [32,33]. Likewise, the quadripartite “Zero by 30” strategy frames mass dog vaccination, access to human post-exposure prophylaxis, and community engagement as the combined pathway to ending human deaths from dog-mediated rabies [7,34], with real-world programs (e.g., Bangladesh) demonstrating sharp declines in human rabies deaths under integrated One Health campaigns [35,36]. Accordingly, throughout this review we treat preventive immunology in livestock as a public-health intervention, making explicit the livestock–environment–human links that inform surveillance, policy, and measurable human outcomes (e.g., incidence, time-to-diagnosis, DALYs averted) [31].

**Figure 1:**
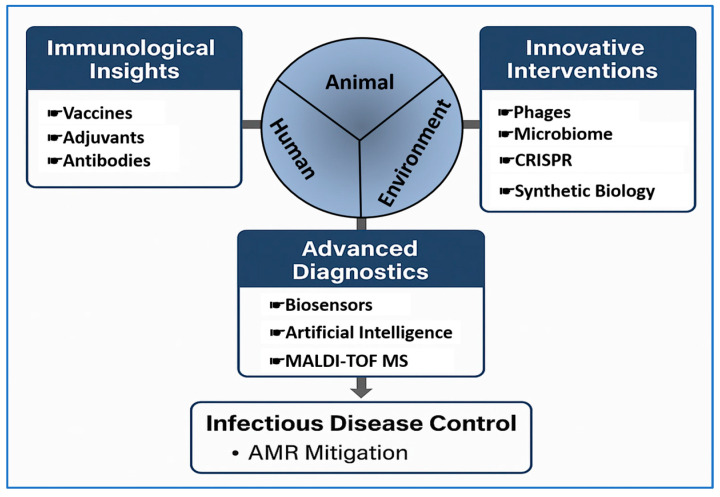
Conceptual framework of preventive immunology in the One Health era. At the center is the One Health interface, showing the close connection between animals, humans, and the environment. Around this core are three main areas driving prevention: immunological insights such as vaccines, adjuvants, and antibodies; innovative interventions including phages, microbiome modulation, CRISPR, and synthetic biology; and advanced diagnostics like biosensors, AI, and MALDI-TOF MS. These approaches strengthen infectious disease prevention, reduce the need for antimicrobials, and help curb AMR. The figure highlights the shift from treatment-focused models toward proactive, prevention-driven strategies that reflect the One Health vision.

The objective of this narrative review is to synthesize and critically evaluate recent advances in preventive immunology, focusing on mechanistic insights, vaccine innovations, complementary interventions (including antibodies, phages, and microbiome approaches), diagnostic technologies, and forward-looking genetic and synthetic biology tools. Framed within a One Health perspective, the review highlights translational opportunities, identifies implementation challenges, and proposes a roadmap for integrating innovative preventive strategies into livestock health management and global health systems (Figure 1). For easy navigation, the review follows four parts: immunological foundations; preventive tools covering vaccines and nonvaccine biologics; diagnostics and surveillance; and policy, governance, and One Health implementation.

## 2. Immunological Foundations of Preventive Immunology

### 2.1. Innate and Adaptive Immunity

Effective prevention depends on the partnership between innate defenses and adaptive memory. Innate responses act first through pattern recognition, then adaptive B- and T-cell immunity provides antigen specificity, durability, and recall. Contemporary reviews summarize how adjuvants and vaccine formulations leverage these layers to achieve lasting protection [37].

Innate immunity forms the first barrier: skin and mucosa, soluble mediators (complement, antimicrobial peptides), and rapid recruitment of phagocytes. These cells express pattern-recognition receptors (PRRs), including Toll-like receptors (TLRs), that sense pathogen-associated patterns and trigger inflammation. TLR stimulation can also induce trained immunity through epigenetic and metabolic reprogramming across animal species [38]. Species differences matter; for example, poultry show distinct TLR regulation during gastrointestinal infection, with consequences for vaccine responsiveness compared with mammals [39].

Adaptive immunity adds specificity and memory. Antigen-presenting cells (dendritic cells, macrophages) process antigens and present peptides via MHC to activate naïve CD4^+^ helper and CD8^+^ cytotoxic T cells. CD4^+^ cells help B cells differentiate into plasma cells that secrete antigen-specific antibodies, while CD8^+^ cells eliminate infected host cells—key for controlling intracellular pathogens [40]. Antibodies neutralize extracellular microbes and toxins. Memory then maintains protection: germinal-center-derived memory B cells enable rapid secondary antibody responses [41], and memory T cells (central and effector) persist in lymphoid and peripheral tissues with epigenetic programs that support swift cytokine production and recall [42].

Species-specific features should guide preventive design. Ruminants are “γδ-high,” with γδ T cells comprising 15–60% of circulating lymphocytes versus 1–5% in humans [43]. These cells contribute to antimicrobial and regulatory functions at mucosal and peripheral sites and can display trained-immunity–like behavior. After BCG vaccination, bovine γδ T cells show epigenetic remodeling with greater chromatin accessibility at innate-response loci and increased IL-6 and TNF-α production [44,45]. This underscores their relevance for livestock vaccines and the value of tailoring strategies to each species’ immune architecture.

In brief, Innate defenses provide fast pattern-based protection; adaptive responses add specificity and memory. Species differences such as the high γδ T-cell compartment in ruminants and distinct TLR regulation in birds shape vaccine performance. Designing preventive tools that align with these features improves durability, breadth, and real-world effectiveness.

### 2.2. Species Differences Matter

Species-level differences in immune architecture regulate vaccine responsiveness. In birds, the bursa of Fabricius is essential for B-cell ontogeny and humoral competence, serving as the primary lymphoid organ for B-cell maturation, an architecture absent in mammals [46]. Swine show strong immunologic and physiologic conservation with humans, making them widely used translational models for infection and vaccinology [47,48]. The porcine immune system mirrors the human system in more than 80% of examined parameters, far exceeding the ~10% overlap seen with mice, and exhibits similar lymphoid organization, cytokine networks, and immune cell marker homology [47,48].

### 2.3. Pathogen Immune Evasion Guides Prevention

Veterinary pathogens are proficient immune manipulators. *Brucella* spp. subvert macrophage function and antigen presentation, undermining sterilizing immunity and hindering vaccination [49,50]. Recent data indicate that *Brucella* modulates the unfolded protein response (UPR) to drive degradation of BLOS1, an important lysosomal-trafficking regulator, further disrupting antigen processing and autophagy in infected macrophages [51]. Smooth LPS also promotes lipid-raft–dependent entry, enabling evasion of lysosomal fusion and fostering intracellular persistence.

*Staphylococcus aureus* is a master immune evader. Protein A and complement-interacting proteins (e.g., SdrE) undermine opsonization and B-cell responses, directly informing vaccine development [52,53]. Protein A binds the Fc region of immunoglobulins, suppressing phagocytosis and skewing antibody orientation to evade clearance [54]. Microbial surface components recognizing adhesive matrix molecules (MSCRAMMs), such as ClfA and fibronectin-binding proteins, increase adhesion and further impede immune targeting [55]. Consequently, *S. aureus* compromises both innate and adaptive immunity, complicating vaccine development [56,57].

In infection with *Mycobacterium bovis* (bovine tuberculosis, bTB), Th1-polarized cellular immunity and granulomatous responses predominate [58]. Protection relies on IFN-γ–mediated macrophage activation and Th1 immunity, but persistent granulomas impede sterilizing clearance and cure [59]. Although CD4^+^ T cells and NK cells assist IFN-γ–dependent containment, limited macrophage responsiveness exacerbates challenges to long-term clearance [60].

### 2.4. Correlates of Protection and T-Helper Polarization

Neutralizing antibodies determine protection for many viral infections, whereas durable control of intracellular bacteria typically depends on Th1-biased cellular immunity (TNF-α and IFN-γ). Enteric and respiratory pathogens often require Th17 and mucosal pathways. These priorities guide current bTB and *Brucella* immunology studies and vaccine design frameworks that leverage adjuvant–PRR crosstalk to shape T-cell fate [37,58].

Across veterinary and human vaccinology, correlates of protection (CoPs) are quantitative immune readouts that predict clinical protection. Recent reviews emphasize the need for robust CoPs for TB/bTB and other intracellular pathogens and outline frameworks and sampling strategies for their validation [61,62]. For *Brucella* spp. and *M. bovis* (bTB), protective immunity is consistently associated with Th1-biased cellular responses—particularly IFN-γ–producing CD4^+^ T cells—supported by macrophage activation, granuloma containment, and complementary biomarkers such as IP-10 and antigen-specific T-cell phenotyping [50,63,64,65,66,67].

At mucosal surfaces, the principal portals of entry for enteric and respiratory pathogens, Th17-associated mechanisms and tissue-resident memory (T_RM) populations sustain barrier immunity and rapid recall. IL-1R–driven adjuvanting and coordinated Th17/T_RM programming are increasingly highlighted as levers for durable mucosal protection [68,69,70]. Adjuvant–PRR crosstalk remains central to T-helper fate: strategic use of TLR and other PRR agonists can bias toward Th1 or Th17, enhance potency, and broaden response breadth [71,72]. Defining pathway-specific CoPs and pairing them with rational adjuvantation provides a mechanistic roadmap for next-generation veterinary vaccines [62].

### 2.5. Adjuvants and PRR Agonists

Contemporary veterinary vaccines increasingly use focused innate stimulation to direct adaptive immunity. TLR agonists—most notably CpG oligodeoxynucleotides (TLR9 in mammals; TLR21 in chickens)—are among the best characterized adjuvants and enhance both humoral and cellular responses [71]. Other PRR ligands similarly bias T-helper phenotypes. Polyinosinic:polycytidylic acid (poly I:C, a TLR3 agonist) promotes antiviral Th1 responses in pigs and cattle, while monophosphoryl lipid A (MPLA, a TLR4 agonist) is widely incorporated into combination adjuvants to strengthen balanced immunity [73,74].

Species-specific applications illustrate this versatility. In chickens, CpG ODNs enhance protection against *Salmonella enterica* and avian influenza [75]. In ruminants, CpG-adjuvanted foot-and-mouth disease vaccines increase IFN-γ responses and broaden cross-serotype protection [71]. Nanotechnology-based adjuvants co-deliver antigens and ligands with controlled spatiotemporal presentation, enabling mucosal targeting and precise immune programming [76]. Aligning adjuvant choice with pathogen biology—Th1 for intracellular pathogens, Th17 for mucosal pathogens, or balanced humoral responses for viral threats—supports systematic optimization of vaccine design.

### 2.6. Mucosal Immunity and Delivery

Because many veterinary pathogens enter through the respiratory or gastrointestinal mucosa, inducing local secretory IgA and tissue-resident T cells is pivotal. Intranasal or oral immunization can elicit pathogen-specific secretory IgA and promote effector homing to mucosal sites, enabling early interception of pathogens [77,78]. T_RM cells (CD69^+^, CD103^+^) persist within mucosal epithelium and provide rapid, localized responses upon re-exposure [79,80]. Next-generation delivery platforms—including viral vectors, extracellular vesicles, and nanoparticles—are being engineered to enhance mucosal immunogenicity [77,81].

### 2.7. Maternal and Early-Life Immunity

In ruminants, neonates depend on colostrum-derived IgG and bioactive factors for protection during the critical early-life window. High-quality colostrum predicts improved growth and reduced disease risk [82,83,84]. Maternal antibodies typically wane by 2–3 months of age, creating a vaccination window that should be carefully integrated into herd health programs [85,86,87,88].

### 2.8. Single-Cell and Systems Immunology in Livestock

Single-cell atlases and multi-omics platforms now provide unprecedented resolution of bovine immune diversity, establishing biomarkers and correlates of protection relevant to disease resilience and vaccine development [66,89]. A landmark Cattle Cell Atlas cataloged more than 1.79 million single cells across 59 tissues, delineating 131 cell types and mapping intercellular networks, transcription-factor programs, and tissue-specific signatures—foundational resources for immunogenomics and precision breeding.

Applied studies demonstrate translational value. In Holstein dairy cows with chronic *S. aureus* mastitis, single-cell RNA-seq identified granulocyte clusters with distinct transcriptional programs linked to migration, differentiation, and inflammation, underscoring their role in pathogenesis and as potential intervention targets [90]. Similarly, in Angus cattle, single-cell transcriptomics of peripheral blood mononuclear cells associated elevated CD8^+^ γδ T cells and pro-inflammatory myeloid activity with stronger immune responsiveness, yielding markers predictive of vaccine efficacy and disease resilience [89]. Beyond cattle, systematic reviews show that single-cell methodologies reveal rare immune subsets, define developmental trajectories, and characterize intercellular communication across livestock species [91].

**Figure 2:**
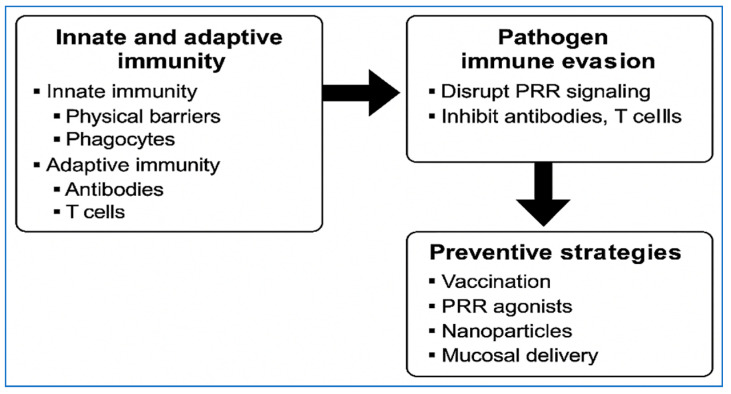
Conceptual overview of immunological foundations guiding preventive strategies. The figure illustrates how innate and adaptive immunity provide the primary defense layers, how pathogens evade these mechanisms through immune disruption, and how this knowledge informs preventive approaches such as vaccination, PRR-based adjuvants, nanotechnology, and mucosal delivery.

Integrated with species-specific features, pathogen evasion mechanisms, correlates of protection, and adjuvant logic, these insights provide the mechanistic basis for next-generation preventive approaches (Table 1; Figure 2). They shape the platform choices discussed below, linking pathogen biology and immune objectives to real-world deployment constraints such as cost, cold chain, and manufacturability. We therefore move from “what immunity is required” to “which vaccines can provide it”.

### 2.9. Zoonotic Transmission Pathways and Human Health Implications

Zoonotic transmission from livestock to people concentrates at predictable touchpoints. Direct contact and occupational exposure place veterinarians, abattoir workers, farmers, and laboratory staff at elevated risk for brucellosis [110,111]. Foodborne pathways remain dominant worldwide: Shiga toxin–producing *E. coli* (STEC), *Campylobacter* spp., and non-typhoidal *Salmonella* (NTS) account for a large share of gastroenteritis, with STEC a well-known precursor of hemolytic uremic syndrome (HUS) [112,113,114,115]. Respiratory/airborne exposure at human–swine and human–poultry interfaces enables zoonotic influenza A infections during farm work, live-market activity, culling, and fair settings [116,117]. Vector-borne and environmental routes also matter: manure runoff and flooding can contaminate surface waters, sustaining environmental reservoirs of enteric pathogens and seeding waterborne outbreaks [118,119].

These interfaces translate into clinically meaningful—and sometimes severe—human disease. Brucellosis and Q fever (*Coxiella burnetii*) often present with febrile illness and, during pregnancy, have been linked to miscarriage and preterm birth [120,121,122]. Foodborne STEC causes gastroenteritis and HUS, a leading cause of pediatric acute kidney injury [113]. *Campylobacter* spp. and NTS contribute substantially to global diarrheal disease and DALYs, particularly among children [114,115]. At animal–human interfaces, zoonotic influenza can progress to pneumonia and occasionally severe outcomes; repeated spillover underscores the need for occupational protection and early detection [116,117].

Animal-side prevention measurably reduces human risk. Targeted vaccination—for example, Rev-1 immunization of small ruminants against *Brucella melitensis*—has been associated with declines in human brucellosis, whereas program lapses have coincided with rebounds in animal and human disease [123,124]. Strengthened biosecurity and hygiene along the food chain, notably Campylobacter control in poultry, have produced population-level reductions in human campylobacteriosis where implemented at scale [125,126]. Rapid diagnostics (e.g., POCT/CRISPR, MALDI-TOF) and integrated wastewater/environmental surveillance provide early, population-level signals that trigger coordinated veterinary and public-health responses, aligning with WHO’s 2024 Wastewater and Environmental Surveillance guidance and recent One Health surveillance roadmaps [127,128]. Finally, AMR stewardship—pairing vaccination (to prevent infections that would otherwise require antibiotics) with prudent antimicrobial use—helps curb cross-sector resistance and protect human health [129].

## 3. Vaccine Innovations: From Classical to Next-Generation Approaches

In this section, we compare classical and next-generation vaccine platforms not only on immunogenicity but also on field-readiness and program constraints (Table 2), setting up Section 4, which extends prevention beyond vaccines. To orient the reader at a glance, Table 2 highlights platform exemplars, program maturity (field readiness), cold-chain needs, and key limitations, with cross-references to delivery and thermostability (Section 3.5).

### 3.1. Classical Platforms and DIVA Logic

Live-attenuated and inactivated vaccines remain cornerstones of veterinary medicine, but marker vaccines—commonly termed DIVA (Differentiating Infected from Vaccinated Animals)—have been transformative. By enabling vaccination without compromising serological surveillance, they preserve eradication credibility. In cattle, gE-deleted BoHV-1 (infectious bovine rhinotracheitis) vaccines are widely used and field-proven, though they do not fully block latency or reactivation, which continues to motivate improved designs [130,131]. In swine, gE/TK-deleted pseudorabies vaccines similarly combine strong protection with DIVA-compliant serology [132,133,134,135]. Collectively, gE-deleted herpesvirus vaccines illustrate how rational deletions sustain disease control while maintaining surveillance integrity [136]. Notably, gE-deleted BoHV-1 DIVA vaccination underpinned successful IBR eradication efforts in parts of Europe and remains the only mandated option in several jurisdictions, highlighting its surveillance value but also the need to address residual latency/reactivation risks [130,131,137]. In comparative terms, classical/DIVA platforms are the most field-mature and programmatically reliable for cattle eradication schemes [137,138], yet they still require management of latent carriers and slower antigenic updating relative to newer platforms. Bottom line: DIVA vaccines remain the default choice for eradication programs that depend on credible sero-surveillance, while latency and slower updating are the main trade-offs (Table 2).

### 3.2. Recombinant Viral Vectors (Cell-Mediated Immunity on Demand)

Recombinant viral vectors—based on adenovirus, poxvirus, or herpesvirus backbones—offer rapid antigen exchange and potent cellular immunity. Multi-gene-deleted herpesvirus vectors, for instance, have been engineered not only for DIVA compatibility but also to reduce latency and reactivation, addressing a long-standing safety concern [132,139,140]. Some designs accommodate heterologous antigen expression, raising prospects for multivalent vaccines that deliver protection against several pathogens in a single dose—an especially practical solution where compliance and logistics are challenging. Recent multivalent designs show that a single product can combine deleted-herpesvirus backbones with antigens from multiple pathogens, reducing doses and improving compliance in the field. Yet, field deployment still hinges on genetic stability, reactivation control, and cost-effective manufacturing [141]. Compared with classical vaccines, viral vectors offer faster re-engineering and stronger T-cell responses, but scale-up is constrained by genetic stability (for herpes backbones) and by manufacturing cost/complexity; field use exists for specific backbones, while broader multi-pathogen licensing remains selective [139,142,143,144]. Program signal: choose vectors when rapid re-targeting and strong T-cell responses are priorities, but plan for genetic-stability QC and costed manufacturing pathways (Table 2).

### 3.3. Nucleic-Acid Platforms (mRNA, DNA) in Livestock

mRNA vaccines are emerging as strong candidates in veterinary medicine, with lipid-nanoparticle (LNP)–encapsulated CSFV E2 mRNA demonstrating robust antibody responses and protection in pigs [145,146,147]. Circular mRNA formats further improve antigen stability and durability, with encouraging results against both CSFV and PRV [148]. Multiple recent studies—including single-dose and circular mRNA formats—report durable protection and improved stability against CSFV (and prototype data for PRV), underscoring fast design cycles and strong immunogenicity in swine [14,145,146]. By contrast, no mRNA vaccines are yet licensed for cattle, reflecting translational and regulatory gaps despite rapid progress. DNA vaccines, though often less immunogenic alone, are increasingly used in heterologous prime–boost regimens (e.g., DNA prime followed by protein or mRNA boost) to diversify responses. Critically, veterinary mRNA is at an early field-validation/TRL stage: it enables rapid antigen design/updates but faces cold-chain/thermostability, GMP fill-finish, and per-dose cost hurdles for food-animal deployment [145,146,148]. Use-case summary: mRNA is ideal when rapid antigenic updates and prototype-to-pilot timelines matter; near-term deployment depends on thermostability solutions and cost controls (Table 2).

### 3.4. Nanovaccines as Smart Adjuvants

As described in Section 2.6, nanotechnology platforms improve antigen stability, uptake, and storage. Their most recent advance is functioning as “smart adjuvants”: co-delivering antigens with innate immune agonists or cytokine cues to program precise immune outcomes [17,76]. By ensuring both signals reach the same antigen-presenting cells, nanoparticles can enhance potency while reducing systemic reactogenicity. Formulation variables—particle size, charge, and epitope repetitiveness—strongly influence T-helper polarization and immune breadth [17]. Moreover, optimized designs now target mucosal surfaces, inducing robust secretory IgA responses in respiratory and intestinal tissues, a feature especially valuable for zoonotic and food-borne pathogen control. Emerging veterinary studies also suggest dose-sparing and improved antigen stability at ambient conditions, but manufacturing complexity and cost remain limiting for large-scale programs in LMICs [14]. Relative to mRNA and vectors, nanovaccines excel at antigen–adjuvant co-localization and mucosal targeting, yet manufacturing complexity, cost, and regulatory familiarity are current bottlenecks for routine field use [149]. Practical takeaway: nanovaccines are strong candidates for mucosal targeting and dose-sparing; plan early for scale-up and regulatory familiarity (Table 2).

### 3.5. Thermostability and Delivery for Low-Resource Settings

Cold-chain dependence remains a major barrier to vaccine access. Thermostable Newcastle disease (NDV) vaccines, derived from the I-2 lineage, have shown strong efficacy under ambient conditions and are widely deployed in low- and middle-income country (LMIC) poultry systems in tablet and ocular formats [150,151,152]. Recent process innovations such as vacuum foam drying (VFD) have further extended NDV shelf-life and heat tolerance from lab- to pilot-scale, illustrating a generalizable path to cold-chain independence [153,154]. Delivery innovations also improve field practicality: microneedle patches deliver antigens to skin-resident antigen-presenting cells while maintaining ambient stability [153], Delivery innovations also improve field practicality: microneedle patches deliver antigens to skin-resident antigen-presenting cells while maintaining ambient stability [149,155], and oral rabies vaccination of wildlife and free-roaming dogs has become a cornerstone of WHO/WOAH strategies for achieving the “Zero by 30” target. Microneedle systems in particular show month-scale room-temperature stability with simple, minimally trained application—an attractive profile for mass campaigns in resource-limited settings [156]. In comparative terms, I-2 NDV is programmatically mature for LMIC poultry, whereas VFD and microneedles are in translation/scale-up: promising thermostability [153] and up to ~12-month room-temperature stability for patches [156,157], but with pending device logistics and unit-cost solutions for routine programs. Equity signal: thermostable formulations and simple delivery (microneedles, oral baits) are the fastest route to scale in LMIC programs; they also de-risk newer platforms by relaxing the cold-chain (Table 2).

### 3.6. Data-Assisted, Systems-Vaccinology Design (Smarter, Faster Pipelines)

Vaccine design pipelines are accelerating through structure-aware epitope prediction, graph-based and transformer models, and prospective validation using single-cell and multi-omics readouts. Tools such as GraphBepi leverage protein-structure constraints to improve B-cell epitope prediction [158,159], while related methods are advancing T-cell epitope discovery and correlates-of-protection analysis, with early veterinary applications [160]. In parallel, single-cell and multi-omics atlases provide biomarker sets for candidate evaluation [161]. Recent work in swine and avian influenza illustrates how computational pipelines can generate pan-strain or broadly protective candidates [162]. Updated reviews in 2025 highlight rapid gains in graph neural networks and transformers, with several immunogens designed prospectively and validated in animal models, including swine influenza [162,163,164]. Compared with empirical screening, these pipelines compress antigen-selection cycles and prioritize cross-protective designs, but require external-validation standards and prospective animal-to-field translation to prove generalizability [158,162]. Implementation note: computational triage shortens design-to-trial timelines; standardized, external benchmarks are needed before broad field claims (Table 2).

### 3.7. Practical Gaps and What to Watch

Despite rapid progress, key hurdles remain. For herpesviruses, latency and reactivation still constrain long-term safety, despite multi-gene deletions [131]. For nucleic-acid platforms, proof-of-concept is strong in pigs, but licensing, GMP-scale production, and distribution logistics remain unresolved for food-producing animals [148]. Nanovaccines and smart adjuvants offer precision but currently face higher production costs compared with classical platforms, making scalability and equity central concerns. Thermostability and novel delivery systems—such as microneedle patches and oral formulations—will be decisive for equitable access in LMICs and for mass campaigns in poultry, wildlife, and free-roaming dogs. Finally, harmonized international regulation will be essential to translate experimental advances into deployable products.

Practically, platform choice should be guided by the immune goal (neutralizing antibody vs. Th1/Th17/TRM; systemic vs. mucosal), program logistics (cold-chain, dose complexity, device supply), and total cost-to-impact—not novelty alone; near-term decisions should track TRL, ambient stability, and effectiveness in target species [153,165]. In summary, vaccine innovations are reshaping veterinary immunology, from DIVA-enabled eradication campaigns to mRNA, nanovaccines, thermostable platforms, and data-assisted design. Table 2 has been updated to show “what to use when”, emphasizing readiness, logistics, and equity alongside immunogenicity.

**Table 2:** Overview of veterinary vaccine platforms—readiness, logistics, and key considerations.

Platform	Examples/Typical Use	Main Benefits	Field Readiness	Cold-Chain and Delivery	Key Constraints and Considerations	References
Classical and DIVA vaccines	gE-deleted BoHV-1 for IBR; gE/TK-deleted pseudorabies in swine	Well understood; reliable protection; compatible with surveillance (DIVA)	Widely used; licensed for eradication programs	Standard refrigeration; often multiple doses	Latent infection/reactivation in herpesviruses; slower to update antigens; DIVA testing requires planning	[132]
Recombinant viral vectors	Adenovirus, poxvirus, multi-gene-deleted herpesvirus; some multivalent designs	Can be retargeted more quickly than classical vaccines; strong T-cell responses	Used for specific backbones; broader licensing still limited	Refrigerated; injection; needs quality checks for genetic stability	Genetic stability must be monitored; reactivation control for herpes backbones; manufacturing can be complex and costly	[132,166]
mRNA and DNA vaccines	LNP-mRNA (e.g., CSFV E2); circular mRNA; DNA prime then protein or mRNA boost	Fast design and updates; flexible prime–boost combinations	Early field stage in livestock (proof of concept in swine; few licenses in food animals)	Usually frozen or refrigerated for mRNA; fill-finish capacity is a bottleneck	Regulatory pathways still developing; needs better thermostability and lower cost per dose; scale-up for GMP manufacturing	[148]
Nanovaccines (as adjuvants or carriers)	Co-delivery of antigen with innate agonists; mucosal targeting; dose-sparing	Improves antigen delivery and can shape the immune response; may allow room-temperature stability in some designs	Early to mid-development; limited field validation so far	May allow ambient stability depending on formulation	Manufacturing and quality control are demanding; cost and regulatory familiarity are current barriers	[146,167]
Thermostable and delivery innovations	NDV I-2 for poultry in LMICs; vacuum-foam drying; microneedle patches	Better stability and simpler use; supports large-scale campaigns	High for NDV I-2; early to mid-development for vacuum-foam drying and microneedles	Ambient-stable candidates; devices (patches) require reliable supply	Need regulatory and supply chain scaling for devices; unit cost must be acceptable for routine programs	[149,165,168]
Oral vaccines	Rabies baits for wildlife and free-roaming dogs	Non-invasive; practical for wide coverage in the field	Established for wildlife/dog programs	Often stable in the field; success depends on distribution and acceptance	Monitor ecological safety and program performance; community acceptance matters	[169]
Computational (data-assisted) design	Structure-aware B- and T-cell epitope selection; graph/transformer models; checked with single-cell readouts	Shortens early selection; helps aim for cross-protective candidates	Early stage (needs more prospective livestock validation)	Not applicable (design step only)	Requires external reference benchmarks and prospective animal-to-field studies before broad claims	[162]

## 4. Beyond Vaccines: Emerging Preventive Immunological Strategies

We now move beyond vaccines to approaches that bridge early protection, reduce shedding, and complement immunization—monoclonals, cytokine/PRR modulators, microbiota interventions, phages, gene editing, and diagnostics—each compared on readiness, advantages, and limitations (Section 4.1, Section 4.2, Section 4.3, Section 4.4, Section 4.5 and Section 4.6).

### 4.1. Monoclonal Antibodies and Passive Immunization

Passive immunotherapies provide immediate, antigen-specific protection and are most useful for outbreak control, neonatal and periweaning windows, and vulnerable groups. Most licensed veterinary monoclonals focus on companion animals, but plant production and species-matched isotypes are lowering costs and extending feasibility to livestock [170]. Compared with vaccination, monoclonals give instant protection and precise targeting but often require repeat dosing, a cold chain, and carry higher per-dose cost; field use in food animals is emerging as manufacturability improves [170]. A plant-produced biosimilar anti-IL-31 (lokivetmab) showed safety and activity, supporting scalable biologic production for food animals [170].

In cattle, bovine respiratory syncytial virus is a major target; antibodies to the G and F proteins now show extended durability with strong protection in calves [7,171,172,173]. Proof of concept for passive anti-BRSV protection is established, but durability, dosing logistics, and interference with active immunization remain constraints [174,175]. In poultry and swine, broadly neutralizing antibodies to conserved influenza epitopes provide complete protection in lethal H5N1 models, and egg-yolk IgY shows scalable protection in vivo [176,177]. Recent studies support antibody-based protection against H5N1 in vivo (IgY) and in large animals/primates for broadly neutralizing antibodies; production scale and breadth against evolving clade 2.3.4.4b viruses must be demonstrated in routine farm settings [177,178,179]. For swine enteric diseases, maternal immunization and porcine-derived monoclonals generated via single-B-cell approaches are advancing, with improved pharmacokinetics and reduced immunogenicity [180,181]. Single-B-cell pipelines now produce porcine monoclonals against enteric targets, marking a shift from heterologous to species-matched therapeutics that improves safety and half-life [181,182].

Key hurdles include cost, repeated dosing, delivery logistics, and regulatory approval in food-producing species. Nevertheless, expanding pipelines, improved Fc designs, and low-cost expression systems are opening options for outbreak prophylaxis, neonatal bridging, and high-value breeder protection [183]. Relative to vaccines and microbiota tools, veterinary monoclonals are at an intermediate stage; adoption will depend on dose-sparing engineering, room-temperature-tolerant formulations, and clear regulatory paths for production animals [170,174]. Monoclonals fit neonatal bridging, outbreak ring prophylaxis, or high-value breeder protection, while vaccination provides durable herd immunity.

### 4.2. Immunomodulators and Cytokine Therapies

Host-directed interventions shorten susceptibility windows, reduce shedding, and complement vaccines [71]. Type III interferons are promising for mucosal protection; recombinant bovine IFN-λ3 shows antiviral effects against BVDV in vitro and in cattle, and pegylated forms are moving into in vivo testing [184]. These responses mirror earlier findings that IFN-λ can protect against foot and mouth disease where rapid innate activity is vital [185,186]. IL-1β adjuvanting in pigs promotes Th17 and tissue-resident memory T cells with broader influenza protection [187].

Compared with monoclonals (immediate but transient) and classic vaccines (durable but slower), cytokine and interferon approaches provide rapid innate amplification and can potentiate vaccines, but they require careful dose control, exposure-modulating formulations, and delivery suited to field use [184,185,186]. Overall, IFN-λ in food animals is translational with robust target-species signals and limited routine deployment. Innate stimulants such as β-glucans prime trained immunity; in calves, supplementation reduces diarrhea and improves health, and poultry studies show enhanced macrophage activity [188]. Controlled studies report reduced diarrhea and lower BRD incidence after early β-glucan stimulation, supporting a low-cost, scalable option for perinatal risk windows, though effects are context- and product-specific and need standardized quality control [188,189,190].

PRR agonists, including TLR and C-type lectin ligands, are advancing as stand-alone immunotherapies; a 2024 review confirms their safety and effectiveness across livestock [71]. Field readiness varies: poultry applications (including in ovo use) are most mature, whereas large-ruminant programs remain early to translational; dual-PRR chimeras (for example, TLR2/7, TLR2/NOD2) improve co-engagement but still face manufacturing and regulatory hurdles [191,192]. Cytokine/PRR strategies are most impactful when paired with vaccination to accelerate onset of protection during high-risk periods.

### 4.3. Probiotics, Prebiotics, and Gut Microbiota Modulation

Microbiota-targeted interventions strengthen gut health, enhance mucosal immunity, and reduce pathogen burden [193]. In calves, multispecies probiotics lower fecal pathogen loads within the first week, and controlled work confirms day-7 reductions in pre-weaned calves [194]. Encapsulated synbiotics in neonatal buffalo calves improve feed intake, growth, and gut health more than single or unencapsulated formats, with optimization studies supporting immune and growth benefits [194,195,196].

Postbiotics offer stable alternatives: in poultry, supplementation enhances mucin secretion, antibody responses, and growth [197]. Reviews and trials indicate postbiotics maintain broiler performance under challenge while providing immunomodulatory effects and a heat-stable, feed-integrated option compared with live probiotics [197,198]. Effects remain strain- and disease-specific: bovine-origin probiotics alleviate rotavirus-induced lesions, and multiple studies show probiotics/IgY can mitigate rotavirus-associated diarrhea; other work modulates immunity without reducing burdens from *Mycobacterium avium* subsp. *paratuberculosis*, emphasizing target matching and endpoint choice [199,200,201,202,203]. Real-world reliability hinges on verified strain identity and viability, appropriate dosing, and heat stability; postbiotics and encapsulation can improve consistency, but standardized QA and clinically meaningful endpoints remain essential [197,198,204].

Compared with vaccines and monoclonals, these tools are lower cost, safer, and easier to scale via feed or water, but effect sizes vary, benefits are strain and formulation dependent, and regulatory categories and quality control differ across countries. Field maturity is moderate for calf scours prevention and advanced for broiler performance; precision consortia and heat-stable encapsulates are moving toward translational testing [205,206]. Use probiotics/synbiotics for perinatal and stress periods, prefer encapsulated or heat-stable formats where supply chains are fragile, and pair with vaccination or cytokine adjuvants to widen protection.

### 4.4. Phage-Based Immunomodulation

Bacteriophages provide targeted antibacterial activity and can modulate host responses. In poultry, swine, and cattle, phage applications reduce *Salmonella* and *Escherichia coli* (*E. coli*) colonization, improve gut health, and support growth [207]. Compared with antibiotics and broad microbiota tools, phages are pathogen-specific and microbiome-sparing, with the strongest field evidence in poultry; dairy applications are growing but remain earlier stage [208,209]. Phages can shape cytokine profiles and macrophage activity; repeated exposure may also trigger immune recognition, so dosing should consider host responses [210,211,212]. Delivery is flexible: in ovo or early-life cocktails reduce *Salmonella* in poultry; intramammary use lowers *Staphylococcus aureus* loads in mastitis models; feed- and water-delivered cocktails scale under farm conditions [208,213,214].

Engineered phages expand host range and reduce resistance; a four-phage cocktail (SNIPR001) outperforms single phages, suppresses *E. coli* in gut models, and is safe in animals, with early clinical testing underway [215,216]. Barriers include host specificity, gut stability, resistance development, and fragmented regulation. There are also practical hurdles: repeated dosing can trigger immune recognition, performance can drop with farm-to-farm environmental differences, and intramammary delivery is not always straightforward; therefore, routine programs should budget for periodic cocktail refresh, run on-farm stability checks, and monitor resistance trends while staying aligned with regulators [164,165,166].

Standardized adaptation protocols and quality by design will determine approval speed and international use [217,218]. Phages align with antimicrobial stewardship by targeting pathogens while sparing commensals; current practice is most mature for poultry food-chain risk reduction, with bovine mastitis programs earlier and engineered phages in translation [219,220,221]. Table 3 summarizes applications, benefits, limitations, and references for natural and engineered phages.

### 4.5. Gene Editing and CRISPR-Based Preventive Approaches

Gene editing can build host resistance to priority pathogens, control viral entry factors, and enable next-generation antimicrobials that disable resistance genes [227,228]. Compared with vaccination and monoclonals, host-factor editing offers durable protection in a single intervention but has higher regulatory and ethical complexity and needs validation across breeds before routine use [229]. The most advanced program targets PRRSV via CD163; edited pigs show robust resistance to infection and disease, including protection against multiple virulent strains where vaccination alone is insufficient [230]. Evidence spans complete resistance in CD163 knockouts and disease resistance without production penalties when editing the SRCR5/PSTII domain, making PRRSV editing a benchmark in food animals [227,231,232]. For avian influenza, chickens carrying ANP32A substitutions that block viral polymerase–host interactions achieve near-complete protection at standard challenge doses; higher doses indicate a need for stacked edits and vaccine pairing [233]. In cattle, CD46 editing reduces susceptibility to bovine viral diarrhea virus, and the first edited calf with altered CD46 residues shows decreased permissiveness in primary cells, with replication across genetic backgrounds underway [234,235,236]. Beyond entry factors, NRAMP1 (SLC11A1) knock-in confers resistance to *Mycobacterium bovis*, supporting macrophage-pathway editing for intracellular pathogens [237].

As a complementary approach, CRISPR delivered by phages can disable antibiotic resistance genes or essential loci with limited microbiome disruption, though success depends on delivery efficiency, escape control, and regulatory acceptance [238]. Engineered CRISPR-armed phage cocktails such as SNIPR001 reduce gut *E. coli* in mice, are well tolerated in minipigs, and have entered early human trials, indicating a translational path for precision antimicrobial tools [215]. In practice, it is still necessary to contend with off-target edits, mosaicism, fitness trade-offs, and the risk of viral adaptation or escape—for example, ANP32A-edited birds can lose protection against AIV under higher challenge doses [233]. Regulatory acceptance also remains uneven and case-by-case across jurisdictions, creating a fragmented path to deployment [229].

Safety, ethics, and regulation remain central. Editing pathogen receptors (CD163, ANP32A, CD46) requires assessment of off-target edits, fitness trade-offs, and viral adaptation risks (Table 4). Mosaicism in livestock embryos should be minimized through editor choice, timing, and screening workflows [239,240]. Traceability systems, welfare assessments, and phased approvals are critical, and current regulatory outlooks are case by case; clear benefit–risk framing and interoperable traceability will accelerate acceptance [229,241]. From a One Health perspective, gene editing could reduce zoonotic spillover and antimicrobial use by preventing primary infections and limiting transmission. In practice, stack edits with vaccination and strong diagnostics to build durable, low-recurring-cost prevention and limit transmission [233].

### 4.6. Diagnostics as Preventive Tools

Diagnostics now support early detection, risk prediction, and targeted intervention at herd and population levels. Point-of-care antigen tests, lateral-flow devices, compact PCR, and field-ready ELISA readers provide rapid results for *Brucella* spp., *Salmonella enterica*, and avian influenza viruses, reducing reliance on centralized laboratories [244,245]. Compared with centralized PCR, current point-of-care methods offer speed and decentralization but need lot-to-lot quality assurance, external validation, and robust sample preparation; antigen formats are most mature, while molecular systems are rapidly maturing and standardizing workflows [246].

In parallel, wastewater and farm-adjacent environmental surveillance turn detection into action—delivering early, population-level signals that complement case-based reports and trigger upstream measures (vaccination, targeted biosecurity, prophylaxis) and faster community alerts [127,128].

Reviews confirm that point-of-care platforms can detect pathogens prior to clinical signs, enabling earlier interventions and reduced antimicrobial use without compromising productivity [247,248]. Examples include a lateral-flow immunoassay for *Brucella* with high sensitivity and specificity, and the Alveo Sense RT-LAMP system validated for H5, H7, and H9 avian influenza in about 45 min from poultry swabs, with laboratory validation reported in 2025 [246,249]. Field readiness for bird-side molecular tests is translational: laboratory validation is published and industry partners are engaged, but multi-site performance and supply reliability still require documentation [246].

Beyond point-of-care tests, plasmonic immunosensors and microfluidic designs can detect multiple viral antigens with high sensitivity [250,251]. Wearables and indwelling sensors, including accelerometers, rumen boluses, and infrared thermography, track temperature, rumination, and activity; deviations often precede clinical signs [252,253]. Continuous sensors offer earlier risk signals but need cost justification, calibration, and data systems that convert alerts into actions [254,255].

Triangulating animal-health signals with wastewater trends makes the “one sample, many analyses” model real—enabling joint veterinary–public-health decisions that sharpen risk communication, steer resources, and accelerate response; for example, CDC’s influenza A wastewater dashboard strengthened situational awareness for avian-origin H5N1 in 2024 [256,257].

Isothermal methods such as LAMP and RPA shorten turnaround, while CRISPR-based assays add high analytical specificity for pen-side use; constraints include sample preparation, reagent stability, and on-device readouts, which prototypes address with lateral-flow or integrated readers [258,259,260,261]. MALDI-TOF, adapted to veterinary practice, supports rapid mastitis diagnostics and stratification of subclinical disease; studies show high sensitivity and specificity for differentiating disease states and distinguishing contagious from environmental *Streptococcus uberis* strains [262,263,264]. Compared with culture/qPCR, MALDI-TOF with pattern-based analysis provides minutes-to-result phenotyping from raw milk and supports selective dry-cow therapy, but needs generalization across herds, spectral quality assurance, and reference library governance [262,265,266].

Models that integrate milk quality, behavior, and physiology identify metritis and ketosis before clinical onset; precision-livestock systems pair rumen boluses with automated analytics to monitor rumination, temperature, activity, and drinking, producing alerts for heat stress, infection, and welfare risks [267,268,269,270,271,272]. These systems are ready for anomaly detection in commercial collars and boluses, but transparent performance metrics, interoperable data standards, and human-in-the-loop protocols are needed to limit false positives and alert fatigue [254,273].

Challenges include high costs in low- and middle-income settings, limited technical training, regulatory hurdles for novel assays, and data quality and sharing issues. Many CRISPR-based platforms still need validation for field robustness, reagent stability, and supply reliability [274]. Broader One Health assessments highlight persistent gaps in intersectoral governance and harmonized standards for veterinary diagnostics [275]. Near-term program priorities are bird-side RT-LAMP with validated panels, MALDI-TOF-supported mastitis triage, and wearable systems for early alerts, each tied to quality frameworks and cost-effective scale-out [246,254,262]. Operationally, POCT/CRISPR assays, MALDI-TOF + ML, and AI wearables move programs upstream—triggering vaccination, biosecurity, or targeted prophylaxis earlier (Table 5), and feeding into the policy/AMR agenda in Section 7.

### 4.7. Limitations, Failure Cases, and Conflicting Evidence

Across innovative modalities, real-world deployment reveals recurring failure modes. CRISPR-edited livestock can reduce susceptibility—e.g., ANP32 family edits in chickens against avian influenza virus [233] and CD163 domain edits in pigs for PRRSV [230,231,232]—yet unresolved risks include off-target edits, embryo mosaicism, fitness trade-offs, and viral adaptation/escape at higher challenge doses. These observations motivate stacked edits and pairing with vaccination/diagnostics rather than replacement [233]. In cattle, CD46 editing has produced the first calf with reduced susceptibility to BVDV [234,235]. Regulatory pathways remain fragmented internationally and may delay scale-up in food animals [229].

For bacteriophages, strong signals exist in poultry and targeted mastitis models [219,220,222], but narrow host range, on-farm stability, resistance development, immune neutralization with repeated exposure, and heterogeneous regulations can undermine consistency [223,224,225]. Cocktail refresh, standardized adaptation protocols, and quality-by-design manufacturing mitigate but do not eliminate these risks; evidence in dairy is earlier stage, and intramammary delivery presents practical hurdles. Engineered/armed phages are promising yet add cost and regulatory complexity [226].

For probiotics/synbiotics/postbiotics, effects are strain- and indication-specific with variable effect sizes. Trials show cases where immune modulation occurs without meaningful pathogen-load reduction in MAP models, underscoring endpoint selection and quality assurance as critical [203]. Encapsulation and heat-stable postbiotics can improve reliability under farm conditions [197,198,204]. Programs should pre-specify failure-triggering contexts, mitigations (e.g., stacked edits + vaccination; phage cocktail updates; QA-verified strains), and outcome metrics beyond immunogenicity (incidence, shedding, and antimicrobial-use reduction).

## 5. One Health Integration and Environmental Dimensions

### 5.1. Environmental Reservoirs and Spillover Interfaces

Pathogens circulate among wildlife, livestock, people, food systems, water, and soil. Spillover risk rises with land use change, biodiversity loss, climate variability, and trade. Effective prevention requires mapping pathogens, hosts, vectors, and environmental sources at key interfaces such as wildlife–livestock contact zones, live animal markets, and flood-affected waters, and embedding these maps in monitoring and control strategies. The Quadripartite One Health Joint Plan of Action (2022–2026) prioritizes cross-sector prevention through surveillance, vaccination, and risk reduction under a shared FAO–UNEP–WHO–WOAH framework [292]. Reviews still note gaps in wildlife-focused studies, including inconsistent interdisciplinary design, limited stakeholder engagement, and weak integration of environmental data—gaps the OH JPA seeks to address [293].

Mapping wildlife–livestock–human contact zones—including live animal markets and trade corridors—allows programs to anticipate spillover and pre-position countermeasures (targeted vaccination, temporary market closures or “rest days,” enhanced biosecurity, and risk communication), strengthening the human-health link within One Health planning [294,295,296,297].

Case studies illustrate value. Drivers of Zoonotic Viral Spillover shows how land cover change increases contact among humans, livestock, and wildlife, elevating risks for bat coronaviruses, influenza, and henipaviruses [298]. Developing a One Health Data Integration Framework outlines how combining genomic epidemiology with real-time surveillance across animal, human, and environmental systems can detect novel reservoirs earlier and reduce spillover risk [299].

### 5.2. AMR as an Environmental Challenge

Antimicrobial resistance circulates through manure, wastewater, soil, and surface waters, where antibiotics, resistant bacteria, and ARGs co-occur and spread via horizontal gene transfer. Agricultural soils exposed to antibiotics and heavy metals carry elevated ARG burdens, with strong correlations between metal concentrations and ARG prevalence, especially in manure- or effluent-amended soils [300,301]. Quadripartite guidance on integrated AMR/AMU surveillance links resistance in animals with human and environmental data, aligning with the Global Leaders Group and WHO GLASS priorities [4]. Metagenomic studies confirm that co-selection by metals (arsenic, mercury, chromium) and antibiotic residues accelerates ARG spread [301,302]. Urban wastewater analyses further associate ARG abundance with pollution indices, highlighting spillover risks into human water systems [302,303]. Integrated surveillance that combines livestock antimicrobial usage with environmental sampling (soil, water, runoff) and human health outcomes identifies resistance hotspots more effectively than health-sector surveillance alone.

### 5.3. Integrated Surveillance: From Wastewater to Farm Biosecurity

Wastewater-based surveillance has emerged as a cost-efficient, noninvasive early-warning tool that complements clinical and farm monitoring. Pilot studies show utility for zoonoses and AMR, with outputs informing public health actions [304]. Applied to agricultural effluents, it has detected ARGs and zoonotic markers downstream of farms, indicating environmental spillover into human-adjacent systems [129,305]. On farms, pairing rapid diagnostics (point-of-care, isothermal or CRISPR assays) with vaccination and biosecurity accelerates intervention and supports antimicrobial stewardship. Environmental sampling of water, slurry, and dust provides leading indicators of emerging risk. These approaches align with OH JPA priorities for early detection, joint risk assessment, and coordinated response [292]. Increasingly, real-time surveillance architectures integrate environmental data, veterinary diagnostics, and biosensor outputs, linking animal, environmental, and human indicators. Harmonized data-sharing platforms—supported by digital tools, regional AMR consortia, and cross-sector governance—are essential to scale these systems globally [305,306].

### 5.4. Governance, Implementation, and Metrics

Operationalizing One Health requires harmonized validation standards, interoperable data systems, and shared performance metrics across animal, human, and environmental sectors. The OH JPA highlights capacity needs such as laboratory networks, risk communication, and integrated surveillance, with emphasis on country-led implementation supported by regional and global coordination [292]. Key enablers include embedding environmental AMR measures into national surveillance, scaling preventive interventions (vaccines, diagnostics, biosecurity) where environmental exposures sustain endemic risk, securing sustained financing for cross-sector platforms, and transparent reporting that links actions to outcomes such as reduced AMU/AMR, earlier outbreak control, and fewer spillover events [307].

Cross-sector dashboards should report human outcomes alongside animal and environmental indicators to keep One Health accountable—tracking human incidence with AMR/antibiotic-use context, time-to-detection/diagnosis and other timeliness measures, and burden averted (e.g., DALYs) and integrating these with animal-event alerts and environmental signals within systems aligned to the Quadripartite One Health Joint Plan of Action [1]. This shared scorecard follows Tripartite guidance on joint surveillance and routine information-sharing (SIS-OT), leverages standardized AMR/AMU indicators from WHO’s GLASS, links to official animal-health event reporting via WOAH’s WAHIS, and applies established timeliness metrics from the IHR/Joint External Evaluation and recent methodological frameworks—so veterinary, environmental, and public-health teams are acting on the same evidence base [1,307,308].

**Figure 3:**
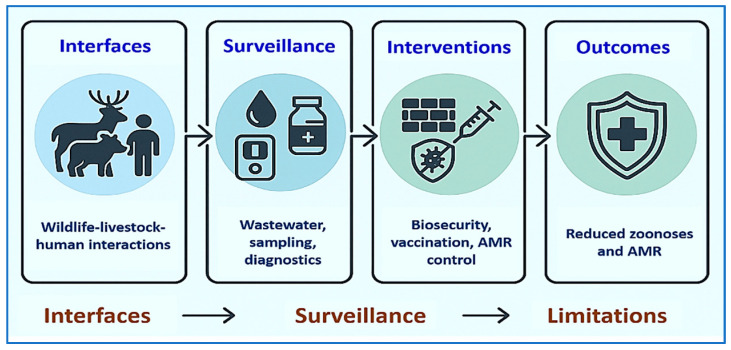
One Health prevention pathway—animal–environment interfaces to public-health outcomes. Arrows show the progression from interfaces (wildlife–livestock–human contact) → surveillance (wastewater, environmental sampling, point-of-care/lab diagnostics) → interventions (biosecurity, targeted vaccination, AMR control) → human-facing outcomes: fewer cases of gastroenteritis/HUS (STEC), febrile illness and adverse pregnancy outcomes (brucellosis, Q fever), and pneumonia (zoonotic influenza), with reduced AMR burden. Bold arrows highlight that animal- and environment-derived signals drive public-health action (community alerts, prophylaxis, resource allocation), linking Section 5.1, Section 5.2, Section 5.3 and Section 5.4 with Section 2.9 and Section 4.6 and closing the loop from animal prevention to measurable gains in human health.

Evidence of progress is emerging. Tegegne et al. applied the OH-EpiCap tool and found that countries with clear governance and data-sharing frameworks reported more transparently [309]. Bengtsson-Palme et al. showed that environmental AMR monitoring can provide early signals of intervention effectiveness [310]. Asaaga et al. documented how cross-sector governance and multi-stakeholder networks in India improved accountability and strengthened One Health reviews [311]. Overall, One Health shifts prevention upstream by managing environmental drivers and cross-species interfaces rather than reacting to outbreaks. Integrated surveillance (including wastewater), environmental AMR monitoring, vaccination, and farm biosecurity—implemented under OH JPA governance—offer an evidence-based pathway to reduce zoonotic spillover and antimicrobial resistance. Figure 3 illustrates this pathway from animal–human–environment interfaces through surveillance to targeted interventions, reinforcing the health of animals, humans, and ecosystems [292].

## 6. Translational and Policy Challenges

Rapid scanning to orient readers may be performed from Table 2 and Table 5 (readiness, use cases, limitations) and Figure 1 and Figure 3 for the One Health approach from prevention to reduced use of antimicrobials.

### 6.1. Regulatory and Approval Pathways

Biotechnologies such as gene editing, CRISPR-based antimicrobials, and newer vaccine platforms (for example, mRNA and thermostable formulations) are advancing faster than many regulatory frameworks. In the United States, FDA guidance (GFI #187A) clarifies oversight of intentional genomic alterations, defines when enforcement discretion may apply, and categorizes products by risk [312]. Complementary draft guidance (GFI #187B) details technical requirements for approval, including molecular characterization, durability, and performance metrics [313]. In January 2025, FDA finalized GFI #187B to sit alongside GFI #187A’s risk-based approach; the Agency also issued low-risk determinations for specific alterations (for example, PRLR “slick” cattle), permitting marketing of animals and derived foods after review [314,315].

Globally, approaches remain fragmented. Wray-Cahen et al. describe policies across Argentina, Japan, the European Union, and the United States, noting that while frameworks are emerging, high compliance costs limit commercialization [316]. Countries that distinguish genome editing from GMOs—especially where edits mirror naturally occurring changes—may accelerate deployment under lighter oversight [317]. In the European Union, the 2018 ECJ ruling (Case C-528/16) confirmed organisms obtained by directed mutagenesis fall under the GMO Directive 2001/18/EC; EFSA’s 2025 opinion concluded current guidance can cover new animal biotechnologies, yet authorization still follows GMO procedures [318,319]. The United Kingdom’s Precision Breeding Act (2023) has been implemented for plants, while ministers have deferred activating provisions for animals pending further evidence and engagement, reflecting welfare-led caution in a high-income setting [320,321]. In LMICs, Kenya’s 2022 and 2025 genome-editing guidelines use case-by-case triggers to determine when edits in plants, animals, and microbes fall under biosafety law versus conventional oversight, reducing uncertainty for research and trials [322].

### 6.2. Economic and Logistical Barriers

High production costs, cold-chain needs, and fragile supply chains remain major obstacles, particularly in LMICs. Next-generation vaccine platforms often require complex formulation, stabilization, and delivery systems that are more expensive than conventional products. Entrican et al. note that dose price, cold or ultra-cold storage, and unreliable distribution networks make mRNA and other novel vaccines difficult to deploy at scale [323]. The WHO’s 2024 mRNA Technology Transfer Hub highlights prohibitive capital costs, infrastructure gaps, and workforce shortages in LMICs [242]. Recent analyses emphasize financing and technology-transfer gaps—fill-finish capacity, GMP utilities, and trained personnel—as primary bottlenecks for regional mRNA production and thermostable lines, even when know-how is shared [242].

Vaccine supply chains also show weaknesses in raw-material procurement, fill-finish capacity, cold storage, and last-mile delivery, causing wastage or reduced potency. Additional challenges include training personnel for novel diagnostics or gene-editing workflows, maintaining quality assurance in decentralized systems, and managing stability at ambient or ultra-low temperatures. These barriers slow the transition from proof of concept to field impact.

### 6.3. Ethical, Societal, and Acceptance Issues

Public acceptance of genomic and other biotechnologies in livestock depends on perceived benefits. Support is consistently higher when applications improve animal welfare (for example, disease resistance and reduced suffering) and lower when framed as productivity gains. Koralesky et al. report greater acceptance of welfare-oriented genetic engineering [324], while McFadden et al. find stronger support when safety data are clear and applications focus on disease prevention [325]. Trust building requires transparency, labeling, risk communication, and inclusive governance. Ethical concerns include off-target effects, welfare and environmental risks, and equitable distribution of benefits.

Surveys show that institutional trust (universities versus industry), clarity of information, and opportunities for public deliberation strongly influence acceptance [326]. Presenting these tools as ethical interventions that protect animals and society—rather than as productivity enhancers—improves legitimacy. Recent studies echo this pattern: the public asks first about animal welfare and oversight, while institutional materials often emphasize regulation—revealing a communication gap that programs should close with clear welfare and benefit narratives [327].

### 6.4. Workforce and Capacity Gaps

Scaling advanced diagnostics, data analytics, and gene editing requires specialized skills across veterinary, regulatory, and bioinformatics domains. Many LMICs face shortages in laboratory infrastructure, continuing education, and field-level training. In Vietnam, 80% of field veterinarians identified surveillance and outbreak investigation as priority needs, with the largest gaps at district and commune levels [328]. Capacity-building programs are responding. The ILRI-led “3rd Generation Genomics and Bioinformatics in Africa” initiative trains researchers in long-read sequencing, library preparation, and bioinformatics [329]. At system level, WOAH’s Performance of Veterinary Services (PVS) Pathway links workforce assessments with laboratory quality management and policy advice, providing a scaffold to integrate new biotechnology workflows into national services [330]. WOAH’s broader PVS tools strengthen capacity through assessments, digital support, and laboratory training [331]. Beyond technical skills, development should include regulatory affairs, ethics, and data governance. Investment in continuing education, cross-disciplinary training, and stronger public laboratories is essential to move innovations from research to field deployment.

### 6.5. Policy Integration and One Health Governance

Policy and governance determine whether veterinary immunology translates into real-world outcomes. The One Health Joint Plan of Action (2022–2026) provides a global framework, but success depends on national adaptation, sustainable financing, and accountability [292]. Shared indicators reduced antimicrobial use and resistance, fewer outbreaks, shorter diagnostic lead times, and higher vaccine coverage allow transparent progress tracking. Linking funding to preventive outcomes can further drive adoption. A 2024 review shows that embedding antimicrobial stewardship into national AMR plans and procurement policies improves outcomes under multisectoral governance [332].

Astbury et al. find that policies integrating environmental, veterinary, and human health dimensions enhance coordination [333]. Aenishaenslin et al. introduce the ISSE framework to evaluate whether One Health surveillance delivers health, economic, and environmental results [334]. Countries engaged with OH-JPA frameworks report clearer financing structures, policy alignment, and monitoring, though major gaps remain in LMICs [30]. Practical steps include legislating AMR-reduction targets, creating accountability bodies, allocating preventive budgets, and strengthening inter-ministerial coordination. Transparent processes and community engagement support legitimacy and equity in high-risk or underserved populations.

As summarized in Table 6, addressing translational and policy challenges requires coordinated progress on regulatory harmonization, economic feasibility, societal acceptance, workforce development, and governance. Scientific innovation alone is not enough—robust policy, equitable capacity building, and cross-sector accountability are essential to achieve measurable One Health outcomes.

## 7. Future Directions and Research Gaps

Major advances in veterinary biotechnology and One Health prevention have not closed key gaps. The practical comparisons and readiness grades in Section 3 and Section 4 (and Table 2 and Table 5) should now guide procurement and policy using simple levers: cost per immunized animal, time to protect, and stability profile, aligning prevention with antimicrobial stewardship and climate resilience.

Over the next 12–36 months, priorities are to deliver externally validated predictive tools that generalize across livestock systems; build climate-aware AMR surveillance that links hydro-climate signals with on-farm sampling and wastewater readouts; establish interoperable One Health data flows with clear latency targets; roll out low-cost, standardized WGS pipelines with complete metadata; and adopt risk-proportionate, trust-building governance frameworks—each aligned with the Quadripartite One Health Joint Plan of Action and related WHO surveillance guidance [1].

### 7.1. AI and ML

Data-driven methods for diagnostics and surveillance show promise in radiographic imaging, cytology, and electronic records, but most models are limited to companion animals and small datasets [25]. External validation across livestock systems is rare, and interpretability remains constrained [336]. Progress will depend on larger, multisite datasets, transparent model architectures, and field validation to ensure reliability.

Over the next 0–18 months, focus on releasing multi-site benchmark datasets with transparent model cards [337], reporting blinded external validation with prespecified metrics such as AUROC and calibration in line with TRIPOD-AI [338], and—when trials are conducted—adhering to CONSORT-AI for complete reporting [339]. From 18 to 36 months, run prospective pilots across diverse production systems, monitor dataset shift and drift, publish generalization gaps with predefined rollback criteria, and report real-world cost per decision.

### 7.2. Climate Change and AMR

Links between climate change and AMR are increasingly evidence based. Global syntheses and multi-region analyses connect warming, heat extremes, and hydrologic variability with higher AMR risks in environmental and clinical settings [340,341,342]. Mechanistic and ecological studies show that higher temperatures can favor resistant taxa and speed the proliferation of ARGs [343]. Hydrologic disturbances add risk: in manure-amended systems, adjacent waters show increased ARG loads and defined dissemination pathways, which intensify with heavy rainfall and inundation [344,345]. Field investigations further demonstrate that flooding mobilizes resistance genes from soils into surface waters, increasing opportunities for horizontal gene transfer [346].

Despite these signals, longitudinal, multi-season studies directly linking climate exposures to AMR outcomes remain limited. Meanwhile, mitigation and monitoring are advancing: wastewater-based AMR surveillance is maturing [347,348], and engineering and bioprocess solutions—from enhanced biological treatment to nature-based sorbents—show promise for lowering ARGs in effluents [349,350,351]. Priority actions now include climate-resilient wastewater upgrades and adaptive on-farm practices (for example, improved manure management and runoff control) to curb climate-amplified AMR risks and support stewardship in livestock production [349,351].

Build early-warning pipelines that link temperature and rainfall anomalies with wastewater and runoff AMR signals; publish clear action thresholds for floods and extreme heat; prioritize flood-prone catchments where hydrologic disturbance mobilizes ARGs; and evaluate climate-resilient wastewater upgrades using shared indicators—ARG reduction, downstream attenuation distance, and stewardship-aligned antimicrobial use—drawing on recent WHO Wastewater & Environmental Surveillance guidance and syntheses of AMR in wastewater [128,348].

### 7.3. Integrated One Health Surveillance

Frameworks for cross-sector data integration exist, but siloed systems, incompatible formats, and the lack of real-time platforms limit implementation. Oltean et al. [299] emphasize the importance of governance and analytics but also note persistent delays. Mediouni et al. show that evaluation tools exist but interoperability and timely data sharing remain insufficient [352]. Future progress requires interoperable standards, predictive dashboards linking veterinary diagnostics with environmental indicators, and strong governance mechanisms.

Adopt common data elements and API-based interoperability across veterinary, environmental, and public-health streams; run shared dashboards with clear latency targets (for example, sample-to-alert ≤ 72 h) and routine data-quality audits; and harmonize indicators and governance using established One Health tools—specifically the Tripartite Surveillance and Information Sharing Operational Tool (SIS-OT) from FAO/WHO/WOAH, and the WHO Tricycle methodology for integrated ESBL-*E. coli* surveillance, which has been implemented and refined in multiple settings [353,354,355].

### 7.4. Whole-Genome Sequencing (WGS)

WGS is transformative for AMR detection but constrained by infrastructure and cost. It can identify known and novel resistance genes, yet veterinary and environmental protocols are underdeveloped, databases incomplete, and workflows costly. Kumari et al. demonstrate Oxford Nanopore sequencing for dairy farm surveillance but note technical challenges [356], while Vallejo-Espín et al. stress that non-human reservoirs remain underrepresented [357]. Low-cost workflows, harmonized databases, and standardized protocols are essential for global accessibility.

Validate low-cost Oxford Nanopore workflows for routine farm and wastewater isolates in both LMIC and HIC settings—building on portable, open-source pipelines demonstrated on dairy-farm AMR surveillance [356] and require MIxS-compliant metadata so genomes are findable and comparable across studies [358]. Publish inter-laboratory proficiency-testing results to document accuracy and reproducibility, in line with surveillance quality frameworks [359], and benchmark genotype–phenotype concordance by pathogen and drug using hybrid or long-read assemblies already trialed on dairy-farm isolates [360].

### 7.5. Socioeconomic and Regulatory Barriers

Innovation is further limited by regulatory uncertainty, high compliance costs, and public trust concerns. GMO legislation can impose heavy burdens on genome-edited animals [316], while opaque policy frameworks and societal skepticism slow adoption [361]. Addressing these gaps requires comparative policy analyses, farmer incentive models, and transparent stakeholder engagement to align governance with societal expectations. Closing research gaps in model validation, climate–AMR dynamics, integrated surveillance, WGS implementation, and regulation is critical to move innovations from proof of concept to scalable and equitable impact across animal, human, and environmental health.

Map risk-proportionate regulatory pathways, drawing on recent precedents for genome-edited livestock such as FDA low-risk determinations; pair these with transparent risk communication and meaningful stakeholder engagement; and track clear adoption metrics—time to authorization, compliance cost per unit, and producer trust—so promising tools move from pilot to practice [362].

## 8. Conclusions

Preventive immunology is rapidly becoming a core pillar of animal infectious disease control within the One Health framework, shifting the focus from reacting to outbreaks to preventing them. This review links how the immune system works with next-generation tools—mRNA and DNA vaccines, viral vectors, and nanovaccines—that aim to boost immunogenicity, thermostability, and delivery. We also examine complementary strategies such as monoclonal antibodies, cytokine modulators, probiotics, bacteriophages, and CRISPR gene editing for their potential to deliver targeted, durable protection. Because early action matters, we spotlight diagnostics as frontline preventive tools, including point-of-care assays, biosensors, MALDI-TOF mass spectrometry, and AI-enabled platforms that support earlier diagnosis, risk prediction, and tailored interventions. Alongside the science, we address translation and policy needs—regulatory alignment, scalable manufacturing, reliable delivery, workforce development, and public trust—with particular attention to equitable access in low- and middle-income countries. Environmental realities—AMR reservoirs and zoonotic spillover interfaces—underscore the urgency of integrated monitoring and cross-sector collaboration. Taken together, coordinating scientific innovation with sound governance, economics, and ethics can cut disease burden, curb antimicrobial resistance, and protect animal, human, and ecosystem health. Still, important limitations persist: gene-editing may require stacked edits plus vaccination; phage programs must manage host range, resistance, stability, and regulatory hurdles; and probiotic/postbiotic benefits remain context-specific. We close with practical mitigations and a simple risk–benefit–readiness framework to guide real-world deployment.

## Figures and Tables

**Table 1:** Key Immunological Foundations and Preventive Implications.

Domain	Key Feature	Species/Pathogen Context	Preventive Implication	References
Innate Immunity	PRR signaling (e.g., TLRs)	Mammals (TLR9), Chickens (TLR21)	Basis for CpG ODN adjuvants; drives trained immunity	[92,93]
	Trained immunity	Bovine γδ T cells	Epigenetic reprogramming after BCG; potential vaccine enhancement	[44,94]
Adaptive Immunity	Long-lived memory B/T cells	All livestock species	Basis for durable vaccine protection	[41,42]
	Th1 bias (IFN-γ, TNF-α)	*M. bovis*	Correlates of protection for intracellular bacteria	[59,95]
	Th17 + mucosal T_RM	Enteric & respiratory pathogens	Critical for mucosal vaccines	[48,96]
Species-Specific Features	γδ T cell abundance (15–60%)	Ruminants	Important targets for vaccine design	[43,97]
	Bursa of Fabricius	Birds (e.g., poultry)	Central to B cell ontogeny	[98]
	Human-like immune system	Pigs (~80% overlap)	Translational model for vaccinology	[99,100]
Pathogen Immune Evasion	UPR manipulation, smooth LPS	*Brucella* spp.	Subverts antigen presentation & autophagy	[51,101]
	Protein A, MSCRAMMs	*S. aureus*	Disrupts opsonization & B cell responses	[102,103]
	Granuloma complexity	*M. bovis*	Limits sterilizing immunity	[59,60]
Adjuvants & Platforms	CpG ODNs, MPLA, Poly I:C	Poultry, ruminants, swine	Direct Th bias; cross-serotype protection	[104,105]
	Nanoparticles (lipid, SAPNs, VLPs)	CSFV, PRV	Improve antigen uptake, stability, dose-sparing	[76,106]
Maternal & Early Life	Colostrum-derived IgG	Neonatal ruminants	Passive transfer; defines vaccination windows	[107,108]
Diagnostics	MALDI-TOF MS, AI, biosensors	Herd-level disease control	Rapid pathogen ID & early vaccination planning	[23]
Systems Immunology	scRNA-seq, cell atlases	Bovine mastitis, TB	Defines biomarkers, precision vaccines	[90,109]

**Table 3:** Comparative overview of natural and engineered bacteriophages in preventive immunology, outlining applications, immunological benefits, and limitations.

Phage Type	Applications	Immunological Benefits	Limitations	References
Natural Phages	Used against *Salmonella*, *E. coli*, and *S. aureus* in poultry, swine, and cattle; mastitis control in dairy systems	Reduce pathogen burden; restore microbiota balance; stimulate innate immune responses (macrophage activation, cytokine release)	Narrow host range; risk of bacterial resistance; limited stability under farm conditions; regulatory hurdles	[220,222,223,224]
Engineered Phages	CRISPR-armed phages targeting resistance genes; synthetic phages for broader coverage and lytic activity	High specificity; overcome resistance mechanisms; potential for multivalent action; lower impact on commensals	Complex design and manufacturing; stability and delivery challenges; higher costs; regulatory uncertainty	[211,215,225,226]

**Table 4:** CRISPR-Based Preventive Strategies in Livestock.

Target Gene/Pathway	Species	Pathogen/Disease	Editing Approach	Preventive Outcome	Reference
CD163 (SRCR5 domain removal)	Pig	PRRSV	CRISPR/Cas9-mediated exon deletion	Pigs resistant to PRRSV infection with no detectable viremia post-challenge	[242]
ANP32A (point substitution)	Chicken	Avian influenza virus (AIV)	CRISPR/Cas9-mediated point mutation	Near-complete resistance to AIV replication in edited chickens	[233]
CD46	Cattle	BVDV	CRISPR/Cas9 knockout	Reduced susceptibility of edited calves to BVDV infection	[234]
NRAMP1 (knock-in)	Cattle	Bovine tuberculosis (*M. bovis*)	CRISPR/Cas9 nickase + HMEJ strategy	Edited cattle showed enhanced resistance to bTB in macrophage assays	[237,243]
Phage-delivered CRISPR antimicrobials	Various livestock-associated bacteria	AMR pathogens	Engineered phages delivering CRISPR/Cas payloads	Targeted removal of antimicrobial resistance genes (ARGs); restored antibiotic sensitivity	[215,238]

**Table 5:** Emerging Diagnostic Tools for preventive Immunology.

Platform	Targets/Use	Sample Type	Turnaround Time	Use Cases	Limitations	References
Lateral Flow Immunoassays (LFIA)	Antigens/antibodies (e.g., FMD, *Brucella*)	Blood, milk, swabs	Minutes	On-farm screening for endemic/zoonotic pathogens	Lower sensitivity than lab-based assays	[276]
Isothermal NAAT (LAMP, RPA, ERA + CRISPR)	Pathogen nucleic acids (e.g., ASFV, avian influenza subtypes H5/H7/H9)	Blood, swabs (oropharyngeal, cloacal), serum	~30–60 min	Field-deployable molecular confirmation; early surveillance; reaction even without perfect lab infrastructure	Risk of contamination (especially when amplification and detection are separated); primer design; sample prep; minimal training required	[277,278,279,280]
CRISPR-based assays (e.g., DETECTR, SHERLOCK)	Viral/bacterial DNA or RNA	Swabs, blood	Under 1 h	Highly specific detection of priority pathogens	Still early-stage validation in livestock	[281,282]
MALDI-TOF MS	Microbial proteins/antibiotic resistance markers	Cultured isolates, direct clinical samples	Minutes to hours	Rapid pathogen ID and AMR profiling	Database limitations, infrastructure needs	[23,283,284,285]
Wearables & Biosensors	Physiological biomarkers (e.g., temp, metabolites)	Animal-attached sensors, saliva, milk	Real-time	Continuous herd health monitoring	Cost, calibration, data integration	[252,286,287]
AI & ML Analytics	Big data from diagnostics, sensors, clinical records	Integrated datasets	Real-time to predictive	Outbreak prediction, precision livestock farming	Data standardization, interpretability	[288,289,290,291]

**Table 6:** Governance Elements, Metrics, and Country Implementation Examples.

Governance Element	Key Metrics/Indicators	Country/Region Example	Reference
Regulatory and Approval Pathways	Number of approvals for gene-edited/vaccine products; time-to-market; clarity of guidance	USA FDA GFI #187A/B; Argentina/Japan policies distinguishing genome editing from GMOs	[312,313,317,318]
Economic and Logistical Barriers	Cost per dose; cold chain reliability; vaccine wastage rates; facility capacity	WHO mRNA Technology Transfer Hub (South Africa); livestock vaccination cost studies in LMICs	[242,324,335,336]
Ethical, Societal, and Acceptance Issues	Public acceptance rates; trust in regulatory bodies; presence of labeling/traceability policies	UK public dialog on gene editing; US consumer surveys on livestock gene editing	[325,326,327]
Workforce and Capacity Gaps	Veterinary staff per 10,000 livestock; training hours per year; retention rates; lab diagnostic capacity	Vietnam field vet training study; ILRI genomics training in Africa; WOAH PVS Pathway	[329,330,332]
Policy Integration and One Health Governance	AMU/AMR reduction rates; outbreak frequency; zoonotic spillover tracking; diagnostic lead times; vaccine coverage	Implementation of OH Joint Plan of Action; Canada ISSE framework; national AMR stewardship plans	[30,333,334,335]

## Data Availability

No new data were created or analyzed in this study. Data sharing is not applicable to this article.
